# Acute Dystonic Reaction in the Upper Extremity Following Anesthesia

**DOI:** 10.7759/cureus.31166

**Published:** 2022-11-06

**Authors:** Mili Patel, Benjamin L Park

**Affiliations:** 1 Medicine, Lake Erie College of Osteopathic Medicine, Erie, USA; 2 Anesthesia, Indiana Regional Medical Center, Indiana, USA

**Keywords:** propofol fentanyl movements, bier block, dystonia upper extremity, acute dystonic reaction, tardive dyskinesia, intravenous regional anesthesia, movement disorder, postoperative movement

## Abstract

We present a case of an 83-year-old female who underwent carpal tunnel release with intravenous regional anesthesia (Bier block) and monitored anesthesia care (MAC). After surgery, the patient developed an abnormal motion of her upper extremity, which was treated as an acute dystonic reaction. Dystonic reactions can occasionally be seen as a post-anesthetic complication, but they are most often associated with antidopaminergic medications. Limbs are rarely affected by dystonic reactions, as they usually affect the head and neck. Acute dystonic reactions can be easily treated with diphenhydramine or benzodiazepines to prevent other extrapyramidal symptoms from occurring. The differential, in this case, was widely varied and inappropriate treatment would have been detrimental to patient care.

## Introduction

Movement disorders can occur anytime during the course of the patient’s hospital stay and the differential diagnosis, for movement disorders, can be quite large and varied. In the post-operative period, movement disorders are less common and often associated with medications used for the treatment of nausea, like metoclopramide. These acute dystonias most often affect the head and neck. The patient, in this case, developed a movement disorder post-operatively and it involved only the operative upper extremity. This patient received relatively few medications that are commonly thought to cause acute dystonia. The patient received lidocaine intravenously as the primary anesthetic along with monitored anesthesia care with propofol as an adjuvant. With the anesthetic that the patient received a large differential diagnosis of acute movement disorders needed to be considered when the patient developed a movement disorder.

## Case presentation

The patient was an 83-year-old female of 76 kg who presented to an ambulatory care facility for elective carpal tunnel release surgery. The patient was on hydrochlorothiazide for hypertension and pantoprazole for well-controlled gastroesophageal reflux disease (GERD). Her past surgical history was a tonsillectomy as a child. She took no illicit substances and had no psychiatric history. The anesthesia team classified her as an American Society of Anesthesia (ASA) class 2 patient. She was given no premedication and was taken to the operating room where standard monitors were applied. She was then given 5cc of 1% lidocaine followed by a low-dose infusion of propofol and 50 ucg of fentanyl. At the same time, the operative arm was prepped for an intravenous regional anesthesia (IVRA). After that the arm was exsanguinated and a double tourniquet inflated to 250 mmHg. The patient was then given 40cc of 0.5% lidocaine. The surgery was performed uneventfully and both tourniquets were deflated after 32 minutes. The propofol infusion was discontinued and the patient was taken to the post-operative care area. The patient’s vital signs were stable and she was arousable as well as conversive. After being in the post-operative care area for 15-20 minutes, the patient developed a rhythmic motion of her operative arm. Since leaving the operating room the patient had received no medications.

The motion of the arm consisted of mostly flexion with slight motion to extend the elbow joint. The elbow was held in flexion with a slight rhythmic extension at a rate of two flexion extensions per second. All vital signs remained unchanged, and the patient was at their baseline mental status asking questions and talking. It was only after the post-anesthesia care unit (PACU) staff attempted to hold the arm down that the patient began to feel anxious, and the frequency of the motion increased. The biceps of the patient did have a hypertonic or spasmed feel to the muscle. The motion continued at rest and with intended action. Otherwise, the patient was alert and oriented in no pain or discomfort and had returned to baseline status.

A differential diagnosis of seizure, anxiety, local anesthesia toxicity, tardive dyskinesia, myoclonus, conversion disorder, and dystonic reaction were all considered. The patient was given 25 mg of diphenhydramine followed by 1 mg of midazolam. The motion of the arm quickly subsided after the administration of diphenhydramine and did not return. The patient remained in PACU for about an hour following this incident and was discharged home uneventfully. No additional testing was ordered for the patient, and they were contacted the following day and reported no additional symptoms.

## Discussion

We report a case of a patient presenting with a new onset acute dystonic reaction as a result of propofol and fentanyl administration. There have been a few reported cases of dystonic reactions caused by propofol, but in these situations, the clinical picture was not complicated by IVRA. There have only been two reported incidences of acute dystonic reactions due to fentanyl administration. In one case study, the dystonic reaction was thought to be a result of multiple anesthetic agents being used in combination. This patient had ocular and mandibular effects of dystonia. The patient was successfully treated with diazepam [1]. In another case study, the patient had a dystonic reaction that affected all four limbs. This patient also was given multiple anesthetic agents in conjunction. For treatment, this patient was given clonidine and midazolam, which both failed to reduce symptoms. This patient was then given paracetamol and ketorolac, which resolved her symptoms [2]. In our case, the patient had an acute tremor that was aggravated when the PACU team held the patient’s hand down. The patient noted that the arm was moving and did attempt to end it herself. This along with a negative psychiatric history suggests that conversion disorder was not the cause of this patient's movement. Administration of diphenhydramine and midazolam relieved her symptoms. Diphenhydramine (50 mg IV) is the preferred drug of choice to treat dystonic reactions. While this can provide moderate relief, lorazepam (0.05-0.10 mg/kg IV) and diazepam (0.1 mg/kg) can both be used to treat these involuntary movements [3]. It is important to note as anesthesia care providers, dystonia affecting the extremities is rarely seen and should be taken very seriously. It is imperative to consider the circumstances under which the dystonia occurs (e.g., if the patient is stressed, if the patient is anxious, etc.). Usually, after administration of diphenhydramine or removal of the offending anesthetic, the dystonia should go away in 24-48 hours [4].

In cases of IVRA, considerations for local anesthetic systemic toxicity (LAST) need always be considered. The incidence of LAST occurs at an incidence of 0.03% [5]. The patient received intravenous lidocaine at a dose of 250 mg. For the IVRA, 200 mg of lidocaine was used and 50 mg was given to provide analgesia for the propofol infusion. The toxic dose of lidocaine without epinephrine for this patient would have been 380 mg. The elderly population is at an increased risk of toxicity due to decreased renal clearance and hepatic metabolism of local anesthetics. It has been recommended that the elderly population have the dose of local anesthetic decreased by 10%. Even with that dose reduction, this patient would have had a toxic dose of 342 mg, which was 92 mg above this patient’s received dose [5]. It is currently recommended that the tourniquet remain inflated for 30 minutes to decrease the chances of LAST. The patient was questioned for other signs and symptoms of LAST including tinnitus, circumoral numbness, and metallic taste. The patient was being closely observed in the PACU and nursing staff was informed of what to observe for progression of LAST symptoms. If the patient had experienced more symptoms or developed a progression, intralipid would have been given starting at 1-1.5 mg/kg.

Myoclonus resulting from the use of a tourniquet for the Bier block was also considered as a cause of this patient's symptoms. It is possible that the tourniquet caused a focal mild ischemia, which could cause a myoclonus of the biceps. While nerve damage can occur with a tourniquet time of 32 minutes, it is less likely in patients without preexisting risk factors, i.e., diabetes, obesity, and positioning. Tourniquet-related nerve damage usually follows tourniquet-related nerve pain. This patient had no evidence of pain in that she did not become hypertensive or tachycardic. Additionally, the patient was not difficult to sedate and did not have increased anesthetic requirements that would suggest tourniquet-related pain. Cellular evidence of tourniquet-related muscle damage can be seen between 30 and 60 minutes, but tissue edema occurs only after 60 minutes [6]. Additionally, myoclonus would be unlikely to have responded to the treatment of diphenhydramine.

Tardive dyskinesia and dystonic reactions on the surface appear to be similar and can be easily confused. Both are characterized by involuntary and irregular movements, usually of the head, neck, or upper extremities. The pathophysiology is associated with central nervous system pathology. Tardive dyskinesia is more often associated with dopamine receptor-blocking agents acting in the basal ganglia, and this occurs after an extended period [7].

Dystonia, on the other hand, is an acute condition caused by an imbalance of the dopaminergic-cholinergic system in the basal ganglia. Dystonic reactions occur more frequently than tardive dyskinesia and are more often associated with drugs with cholinergic activity. Propofol, opioids, and antinausea medications have all been implicated in dystonic reactions [8]. The treatment for tardive dyskinesia and dystonia includes diphenhydramine. Diphenhydramine is a nonspecific antihistamine with significant anticholinergic effects. The anticholinergic effects are related to the suppression of movement and dystonic in both tardive and dystonia. Propofol-related dystonias have been described in all phases of care and have been described as delayed up to 30 minutes after cessation of propofol use [8]. As was the case in this patient the dystonia began after arrival in PACU.

Anxiety was also part of the differential for this patient. While tremors and movements can be associated with anxiety, they are nearly always symmetric in nature, will affect fine motor muscles more than gross motor movements, and worsen in intensity with intention. The movement that this patient demonstrated was unilateral and only affected the upper arm. There was no effect on the fingers or forearm of the affected arm. An essential tremor would affect various parts of the arm and the patient and would have been noticed preoperatively. The frequency of essential tremors is between 4-11 Hz [9].

The slight movement or tremor in this patient was about 2-2.5 Hz and therefore not consistent with an essential tremor or alcohol withdrawal tremor (6-11 Hz), or a parkinsonism tremor. Another tremor type would be a psychogenic tremor. Psychogenic tremors change in intensity and will increase, decrease, and stop in intensity, which was not like the tremor occurring in this patient [9].

Additionally, seizure or a seizure-like disorder was considered, in the differential of this patient. The patient had no seizure history and a new onset seizure at the age of 83 years would be unlikely without CNS pathology. The response to treatment with diphenhydramine also suggests that seizure-like activity, would not cause this patient's movement disorder. Conversion disorder was also suggested but also would be unlikely to be newly manifested in an 83-year-old patient.

## Conclusions

Movement disorders in the post-operative area are uncommon and need to be evaluated appropriately. The differential should include acute dystonic reactions because many of the medications used during anesthesia can cause these types of movement disorders. While acute dystonic reactions are more commonly associated with antidopaminergic and antiemetic medications, they can also occur with medications like propofol and fentanyl, which are commonly used during anesthesia. These disorders can occur anytime during the operative course and even be delayed by 30 minutes following administration. Additionally, while acute dystonic reactions more commonly affect the head and neck, they can present in an extremity also. An acute dystonic reaction is easily treated when the diagnosis is made. Medications for the treatment of these movement disorders include diphenhydramine along with possibly benzodiazepines.
